# Cupping for Treating Pain: A Systematic Review

**DOI:** 10.1093/ecam/nep035

**Published:** 2011-06-23

**Authors:** Jong-In Kim, Myeong Soo Lee, Dong-Hyo Lee, Kate Boddy, Edzard Ernst

**Affiliations:** ^1^Korea Institute of Oriental Medicine, Daejeon 305-811, Republic of Korea; ^2^College of Oriental Medicine, Kyung Hee University, Seoul, Republic of Korea; ^3^Complementary Medicine, Peninsula Medical School, Universities of Exeter & Plymouth, Exeter, UK; ^4^College of Oriental Medicine, Wonkwang University, Hospital, Sanbon, Republic of Korea

## Abstract

The objective of this study was to assess the evidence for or against the effectiveness of cupping as a treatment option for pain. Fourteen databases were searched. Randomized clinical trials (RCTs) testing cupping in patients with pain of any origin were considered. Trials using cupping with or without drawing blood were included, while trials comparing cupping with other treatments of unproven efficacy were excluded. Trials with cupping as concomitant treatment together with other treatments of unproven efficacy were excluded. Trials were also excluded if pain was not a central symptom of the condition. The selection of studies, data extraction and validation were performed independently by three reviewers. Seven RCTs met all the inclusion criteria. Two RCTs suggested significant pain reduction for cupping in low back pain compared with usual care (*P* < .01) and analgesia (*P* < .001). Another two RCTs also showed positive effects of cupping in cancer pain (*P* < .05) and trigeminal neuralgia (*P* < .01) compared with anticancer drugs and analgesics, respectively. Two RCTs reported favorable effects of cupping on pain in brachialgia compared with usual care (*P* = .03) or heat pad (*P* < .001). The other RCT failed to show superior effects of cupping on pain in herpes zoster compared with anti-viral medication (*P* = .065). Currently there are few RCTs testing the effectiveness of cupping in the management of pain. Most of the existing trials are of poor quality. Therefore, more rigorous studies are required before the effectiveness of cupping for the treatment of pain can be determined.

## 1. Introduction

Pain is the most common reason for seeking therapeutic alternatives to conventional medicine [1] and the more severe the pain, the more frequent is the use of such therapies [1, 2]. Frequently used treatments include acupuncture, massage and mind-body therapies [1, 2].

Cupping is a physical treatment used by acupuncturists or other therapists, which utilize a glass or bamboo cup to create suction on the skin over a painful area or acupuncture point [3]. It is mostly used in Asian and Middle Eastern countries and has been claimed to reduce pain as well as a host of other symptoms [4]. There are two types of cupping. Dry cupping pulls the skin into the cup without drawing blood. In wet cupping the skin is lacerated so that blood is drawn into the cup.

A recent systematic review included five trials (two randomized clinical trials (RCTs) and three controlled clinical trials (CCTs)) on the effects of wet cupping on musculoskeletal problems [5]. Its findings suggested that wet cupping is effective for treating low back pain. However, the review lacked a comprehensive search, included language restrictions and only searched a limited number of databases. Another limitation is that all of the trials compared cupping in combination with other therapies with either acupuncture or another type of cupping. Furthermore, this review pooled the results regardless of their design which raises the possibility of biased results. The aim of this systematic review therefore, was to summarize and critically evaluate the evidence for or against the effectiveness of cupping as a singular treatment of pain.

## 2. Methods

### 2.1. Data Sources

The following databases were searched from inception through to January 2009: MEDLINE, AMED, EMBASE, CINAHL, five Korean Medical Databases (Korean Studies Information, DBPIA, Korea Institute of Science and Technology Information, KoreaMed, and Research Information Center for Health Database), four Chinese Medical Databases (China National Knowledge Infracture: China Academic Journal, Century Journal Project, China Doctor/Master Dissertation Full Text DB and China Proceedings Conference Full Text DB) and The Cochrane Library 2008, Issue 4. The search terms used were based on two concepts. First concept included terms for cupping and the other concept included terms for pain. The two concepts were combined using the Boolean operator AND. In the English databases it was unnecessary to use synonyms for cupping as the only term used to describe this therapy is cupping. The term “cupping" would also capture dry cupping, wet cupping, cupping therapy, and so forth. Korean and Chinese terms for cupping and pain were used in the Korean and Chinese databases. We also performed electronic searches of relevant journals (FACT (Focus on Alternative and Complementary Therapies), and Research in Complementary Medicine (Forschende Komplementarmedizin) up to January 2009). Reference lists of all obtained papers were searched in addition. Furthermore, our own personal files were manually searched. Hardcopies of all articles were obtained and read in full.

### 2.2. Study Selection

RCTs testing cupping with or without drawing blood as sole or adjunctive treatment, in patients of either sex or any age diagnosed as having any type of pain and assessing clinically relevant outcomes, were included. The RCTs were included whether placebo controlled or controlled against another active treatment or no treatment. Cupping was defined as pulling the skin into the cup with or without drawing blood for therapeutic. Trials with designs that did not allow an evaluation of efficacy of the test intervention (e.g., by using treatments of unproven efficacy in the control group or comparing two different forms of cupping) were excluded. Trials with cupping as concomitant treatment together with other treatments of unproven efficacy were excluded. Trials published in the forms of dissertation and abstract were included. No language restrictions were imposed.

### 2.3. Data Extraction and Quality Assessment

Hard copies of all articles were obtained and read in full. All articles were read by three independent reviewers (J.-I. K., M . S. L. and D.-H. L.) and data from the articles were validated and extracted according to pre-defined criteria (Table 1). No language limitations were imposed.

Risk of bias was assessed using the Cochrane classification in four criteria: randomization, blinding, withdrawals and allocation concealment [6]. Considering that it is very hard to blind therapists to the use of cupping, we assessed patient and assessor blinding separately. We admitted assessor blinding if pain was assessed by another person (not the patient himself) who did not know the group assignment. Disagreements were resolved by discussion among the three reviewers (J.-I. K., M. S. L. and D.-H. L.). There were no disagreements among the three reviewers about risk of biases.

### 2.4. Data Synthesis

The mean change of outcome measures compared to baseline was used to assess the differences between the intervention groups and the control groups. The mean difference (MD) and 95% confidence intervals (CIs) were calculated using the Cochrane Collaboration's software (Review Manager version 5.0 for Windows, Copenhagen: The Nordic Cochrane Center) for continuous data. For studies with insufficient information, we contacted the primary authors to acquire and verify data where possible. The *χ* ^2^ test was used for statistical analysis for trials which reported response rate (RR) using dBSTAT program (http://www.dbstat.com/).

## 3. Results

### 3.1. Study Description

**Figure 1:**
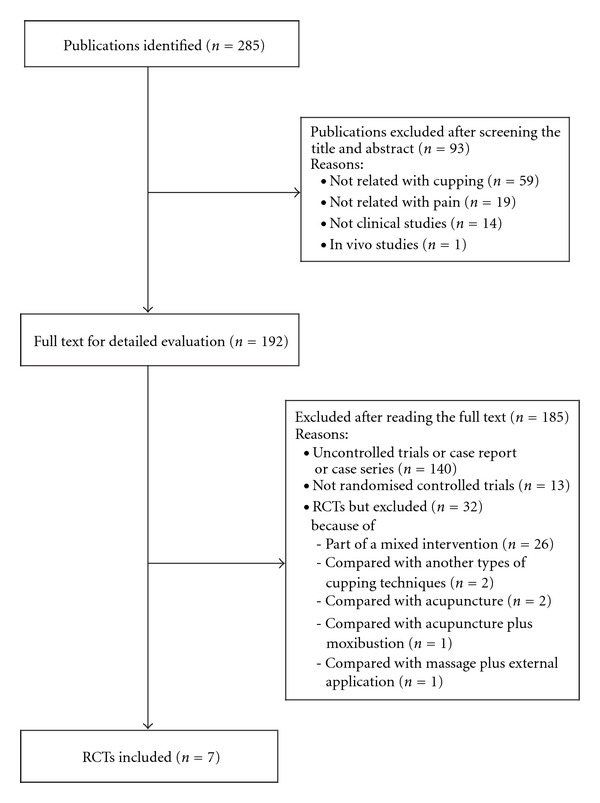
Flowchart of trial selection process. RCT: randomized clinical trial; CCT: non-randomized controlled clinical trial; UCT: uncontrolled clinical trial.

The literature searches revealed 285 articles, of which 278 studies had to be excluded (Figure 1). One hundred and eighty five articles were excluded after retrieving full text and their reasons. Seven RCTs met our inclusion criteria and their key data are listed in Tables 1 and 2 [7–13]. One of the included RCTs originated from Iran [10], four RCTs from China [7–9, 13] and two RCTs from Germany [11, 12]. All of the included trials adopted a two-armed parallel group design. The treated conditions were low back pain [8, 10] cancer pain [7], trigeminal neuralgia [9], Brachialgia paraesthetica nocturna (BPN) [11, 12] and herpes zoster [13]. The subjective outcome measures were the McGill Pain Questionnaire [10], 100 mm visual analogue scales [8, 10, 12], response rate [7–9, 13] and Likert scales [11]. Five trials employed wet cupping [9–13] and two with dry cupping [7, 8]. The number of treatment sessions ranged from one to about nine, with a duration of 5–20 min per session. The rationale for the selection of cupping points was stated in three RCTs to be according to traditional Chinese medicine (TCM) theory [7, 8], clinical experience of expert [9], empirical date [11, 12] or to classical TCM textbook [13]. One RCT followed traditional Iranian Medicine [10]. We contacted the authors for further information about an RCT identified in our searches which was published as proceeding paper [12].

### 3.2. Study Quality

Four RCTs employed the methods of randomization [7, 8, 10, 11] but none adopted both assessor and subject blinding. Assessor blinding was judged to have been achieved in one [12] of the RCTs and three used allocation concealment [10–12]. Sufficient details of drop-outs and withdrawals were described in two RCTs [10, 11].

### 3.3. Outcomes

One RCT [7] compared the effects of dry cupping on cancer pain with conventional drug therapy and reported favorable effects for cupping after 3-day intervention (RR, 67% versus 43%, *P* < .05). Another RCT [8] compared dry cupping with nonsteroidal anti-inflammatory drugs in nonspecific low back pain and suggested a significant difference in pain relief on VAS after treatment duration (MD, 22.8 of 100 mm VAS; 95% CI, 11.4–34.2, *P* < .001). The third RCT [9] suggested that wet cupping reduced pain compared with analgesics in acute trigeminal neuralgia after the intervention period (RR, 93% versus 47%, *P* < .01). The fourth RCT [10] tested wet cupping plus usual care for pain reduction compared with usual care in non-specific low back pain and suggested significant differences in pain relief (McGill Pain Questionnaire) at 3 months after three treatment sessions (MD, 2.2 of 6 points present pain intensity; 95% CI, 1.7–2.6, *P* < .01). The fifth RCT [11] reported that one session of wet cupping plus usual care significantly reduced pain during a week compared with usual care alone in patients with BPN (MD, 1.6 of 10 points score, 95% CI, 0.13–3.07, *P* = .03). The sixth RCT [12] showed favorable effects of one session of wet cupping on pain reduction compared with a heat pad in patients with BPN at 7 days after treatment (MD, 22.9, 100 mm VAS; 95% CI, 10.5–35.3, *P* < .001). A further RCT [13] of wet cupping plus conventional drugs on pain reduction compared with conventional drugs alone in patients with herpes zoster failed to show favorable effects of wet cupping after interventions (RR, 100% versus 88%, *P* = .065).

## 4. Discussion

Few rigorous trials have tested the effects of cupping on pain. The evidence from all RCTs of cupping seems positive. The data suggest effectiveness of cupping compared with conventional treatment [7–9]. Favorable effects were also suggested for wet cupping as an adjunct to conventional drug treatment compared with conventional treatment only [10–13]. None of the reviewed trials reported severe adverse events. The number of trials and the total sample size are too small to distinguish between any nonspecific or specific effects, which preclude any firm conclusions. Moreover, the methodological quality was often poor.

The likelihood of inherent bias in the studies was assessed based on the description of randomization, blinding, withdrawals and allocation concealment. Four of the seven included trials [7–9, 13] had a high risk of bias. Low-quality trials are more likely to overestimate the effect size [14]. Three trials employed allocation concealment [10–12]. Even though blinding patients might be difficult in studies of cupping, specifically wet cupping, assessor blinding can be achieved. One of the RCTs made an attempt to blind assessors. None of the studies used a power calculation, and sample sizes were usually small. In addition, four of the RCTs [7–9, 13] failed to report details about ethical approval. Details of drop-outs and withdrawals were described in two trials [10, 11] and the other RCTs did not report this information which can lead to exclusion or attrition bias. Thus the reliability of the evidence presented here is clearly limited.

Two types of cupping were compared with conventional treatment including drug therapy. Some suggestive evidence of superiority of dry cupping was found compared with conventional drug therapy in patients with low back pain [8] and cancer pain [7]. However, one study failed to compare the baseline values of the outcome measures [7]. Four RCTs compared wet cupping with control treatments [10–13]. One RCT [10] reported favorable effects of wet cupping on pain reduction after 3 months follow up, without assessing it after the intervention period. However, these positive results are not convincing because no information was given about treatment during the 3 months of intervention. Two further RCTs [11, 12] tested the effects of wet cupping on BPN and showed it to be beneficial in the reduction of pain. Even though these RCTs showed significant differences at 7 days after a single session treatment, uncertainty about the effectiveness of single session cupping remains due to lack of follow-up measurements. One trial of wet cupping suggested positive effects of response rate [13], while re-analysis of this results with *χ* ^2^ test failed to do so (*P* = .067). Comparing wet cupping plus usual care (or heat pad) with usual care (or heat pad) [10–13] generated favorable effects on at least one outcome measure. Due to their design (A + B versus B) these RCTs are unable to demonstrate specific therapeutic effects [15]. It is conceivable that with such a design (A + B versus B), the experimental treatment seems effective, even if it is, in fact, a pure placebo: the non-specific effects of A are likely to generate a positive result even in the absence of specific effects of A.

Reports of adverse events with cupping were scarce and those that were reported were mild. Adverse effects of cupping were reported in one [10] of the reviewed RCTs. Three cases of fainting (vaso-vagal syncope) were reported with wet cupping.

Assuming that cupping was beneficial for the management of pain conditions, its mechanisms of action may be of interest. The postulated modes of actions include the interruption of blood circulation and congestion as well as stopping the inflammatory extravasations (escaping of bodily fluids such as blood) from the tissues [3, 4]. Others have postulated that cupping could affect the autonomic nervous system and help to reduce pain [3, 4]. None of these theories are, however, currently established in a scientific sense.

Our review has a number of important limitations. Although strong efforts were made to retrieve all RCTs on the subject, we cannot be absolutely certain that we succeeded. Moreover, selective publishing and reporting are other major causes for bias, which have to be considered. It is conceivable that several negative RCTs remained unpublished and thus distorted the overall picture [16, 17]. Most of the included RCTs that reported positive results come from China, a country which has been shown to produce no negative results [18]. Further limitations include the paucity and the often suboptimal methodological quality of the primary data. One should note, however, that design features such as placebo or blinding are difficult to incorporate in studies of cupping and that research funds are scarce. These are factors that influence both the quality and the quantity of research. In total, these factors limit the conclusiveness of this systematic review.

In conclusion, the results of our systematic review provide some suggestive evidence for the effectiveness of cupping in the management of pain conditions. However, the total number of RCTs included in the analysis and the methodological quality were too low to draw firm conclusions. Future RCTs seem warranted but must overcome the methodological shortcomings of the existing evidence.

## Funding

Korea Institute of Oriental Medicine (K09050) (to J.-I. K., M. S. L. and D.-H. L.).

## Figures and Tables

**Table 1:** Summary of randomized clinical trials of cupping for pain conditions.

First author (year) (ref.), Country	Sample size condition/disease duration	Intervention (regimen)	Control (regimen)	Total session	Pain related main outcome	Main results	Measurement time	Risk of bias
Huang (2006) [7], China	60/cancer pain/5–17 months	(A) Dry-cupping (maintain: 5 min, repeat + maintain: 10–15 min, once a day for 3 days, *n* = 30), plus none	(B) Opioid (Propoxyphene Napsylate and Paracetamol, two tablets, three times daily for 3 days, *n* = 30)	3	(1) Response rate (% reduction of pain ≥ 70%) (2) Pain free duration (h),	(1) A (28/30, 67%); B (13/30, 43%),*P* = .001 (2) A (mean 5.06, range 3–8); B (mean 3.65, range 2–6), *P* < .05	Baseline: before intervention Post: after final intervention	(Y,U,U,U,U)
Hong (2006) [8], China	70/non-specific low back pain/1 week to 3.1 years	(A) Dry cupping (moving cupping, 5 min, 1/2 days for 11 days, *n* = 37), plus none	(B) NSAIDs (Dexibuprofen, 0.15 g, three times daily for 12 days, *n* = 33)	6	(1) Pain on VAS (100 mm) (2) Response rate (% change of VAS ≥ 80%)	(1) Mean difference 22.8 (95% CI, 11.4–34.2), *P* < .0001 in favor of (A) (2) A (21/37, 57%); B (9/33, 27%),*P* = .03	Baseline: before intervention Post: after final intervention	(Y,Y,U,U,U,U)
Zhang (1997) [9], China	45/acute trigeminal neuralgia/6 days to 4 years	(A) Wet-cupping (15 min, once a day for 6 days and then once every other day for three sessions, *n* = 30), plus none	(B) Analgesics (AP-237 50 mg (IM, only first day) and then 60 mg (oral, three times daily for 12 days), *n* = 15)	9	Response rate	A (28/30, 93%); B(7/15, 47%), *P* = .002	Baseline: before intervention Post: after final intervention	(Y,U,U,U,U,U)
Farhadi (2009) [10], Iran	98/non-specific low back pain/≥4 weeks	(A) Wet-cupping (20 min, three stage, 3 days intervals, *n* = 48), plus (B)	(B) Usual care (*n* = 50)	3	PPI of the MPQ (6-point Likert scale)	Mean difference 2.2 points (95% CI, 1.7–2.6), *P* < .01 in favor of (A)	Baseline: before start trial Post: 3 months after final intervention	(Y,Y,Y,U,U,Y)
Lüdtke (2006) [11], Germany	20/BPN/n.r.	(A) Wet-cupping (10 min, once, *n* = 10), plus (B)	(B) Usual care (analgesics, physiotherapies, psychological care, musicotherapy, *n* = 10)	1	Pain in Brachialgia Score (NAS, 10-point Likert scale)	Mean difference 1.6 (95% CI, 0.13–3.07), *P* = .03 in favor of (A)	Baseline: mean 7 days pre-treatment score (days 1–7) Post: mean 7 daily post-treatment score (days 9–15)	(A,A,A,U,U,A)
Michalsen (2007) [12]^a^, Germany	52/BPN/n.r.	(A) Wet-cupping (10 min, once, *n* = 26), plus (B)	(B) Heat pad (*n* = 26)	1	(1) Pain in Brachialgia Score (100 mm VAS) (2) Neck pain (100 mm VAS)	(1) Mean difference 22.9 (95% CI, 10.5–35.3), *P* < .001 in favor of (A) (2) Mean difference 12.6 (95% CI, 6.4–18.8), *P* < .001	Baseline: pre-treatment Post: 7 day after treatment	(Y,Y,U,U,Y,Y)
Xu (2004) [13], China	80/Herpes zoster/1–3 days	(A) Wet-cupping (15 min, once a day for 7 days, *n* = 40), plus (B)	(B) Anti viral (Aciclovir 0.5 g + 5% DW 250 ml (IV), plus Aciclovir cream (external use), two times daily for 7 days, *n* = 40)	7	Response rate (% degree of pain ≥ 60%)	40/40, 100%); B (35/40, 88%), *P* = .065	Baseline: before intervention Post: after final intervention	(Y,U,U,U,U,U)

Risk of bias (randomization, randomization method, drop-out or withdraw, patient blind, assessor blind, allocation concealment). Y: low risk of bias; U: unclear; N: high risk of bias; BPN: Brachialgia paraesthetica nocturna; MPQ: McGill Pain Questionnaire; NAS: Numeric Analogue Scale; n.r.: not reported; NS: not significant; NSAID: non-steroidal anti-inflammatory drug; PPI: Present Pain Intensity; VAS: Visual analogue scale.

^a^The authors were contacted and details were based on information from them.

**Table 2:** Summary of treatment points, their rationales and adverse events.

First author (year) (ref.)	Conditions	Cupping point	Rationales	Adverse effects
Huang (2006) [7]	Cancer pain	Liver cancer: ST36, SP6 or LR14, BL18 Lung cancer: BL13, CV17 or BL15 Large intestine cancer: CV8, BL25 or ST36 Bone Metastases: BL23, ST36, SP6 or Asihyeol (unfixed point) Gastric cancer: CV8, BL21, and so forth.	TCM theory	n.r.
Hong (2006) [8]	Low back pain	Bladder Meridian (BL12–BL27)	TCM theory	n.r.(−)
Zhang (1997) [9]	Acute trigeminal neuralgia	GV14, BL13 (bilateral)	Experience of veteran TCM doctors	n.r.
Farhadi (2008) [10]	Low back pain	Day 0: Between the two scapulas, opposite to T1–T3 Scapular spine Day 3: The sacrum area, between the lumbar vertebrae and the coccyx bone Day 6: The calf area, in the middle surface of gastrocnemius muscle	Traditional Iranian medicine	Vaso-vagal shock (*n* = 3)
Ludtke (2006) [11]	Brachialgia paraesthetica nocturna	The skin at the shoulder triangle (over the Musculus trapezius)	Empirical data	None
Michalsen (2007) [12]^a^	Brachialgia paraesthetica nocturna (neurologically confirmed carpel tunnel syndrome)	The skin at the shoulder triangle (over the Musculus trapezius)	Empirical data	n.r.
Xu (2004) [13]	Herpes zoster	Lesion (the surface of vesicle or erythema and painful place)	TCM theory	n.r.

TCM: Traditional Chinese medicine, n.r.: not reported.

^a^The authors were contacted and details were based on information from them.
